# Supplementary material for: Lineage abundance estimation for SARS-CoV-2 in wastewater using transcriptome quantification techniques
Source: Genome Biol. 2022 Nov 8;23:236. doi: 10.1186/s13059-022-02805-9 (PMC9643916; doi:10.1186/s13059-022-02805-9)
Supplement: Supplementary file 1 — Additional file 1. Includes all supplementary information, supplementary figures and supplementary tables. [file 13059_2022_2805_MOESM1_ESM.pdf]

Lineage abundance estimation for SARS-CoV-2 in wastewater using transcript quantification techniques

Supplementary material

J.A. Baaijens*, A. Zulli*, I.M. Ott*, I. Nika, M.J. van der Lugt, M.E. Petrone, T. Alpert, J.R. Fauver, C.C. Kalinich, C.B.F. Vogel, M.I. Breban, C. Duvallet, K.A. McElroy, N. Ghaeli, M. Imakaev, M.F. Mckenzie-Bennett, K. Robinson, A. Plocik, R. Schilling, M. Pierson, R. Littelfield, M.L. Spencer, B.B. Simen, Yale SARS-CoV-2 Genomic Surveillance Initiative, W.P. Hanage, N.D. Grubaugh†, J. Peccia†, M. Baym†

*Denotes equal contribution
†Denotes co-senior authorship
Author affiliations are listed in main manuscript.

Contents

1 SARS-CoV-2 genomes show sequence diversity within variant lineages 2

2 Prediction noise level per sequence determines the minimal abundance threshold 3

3 Benchmarking results for the VLQ pipeline 4
3.1 Full results for datasets main paper 4
3.2 Impact of sequencing errors on prediction accuracy 5
3.3 The VLQ pipeline can distinguish between highly similar sequences 7

4 Comparing kallisto to other RNA transcript quantification tools 8
4.1 kallisto versus salmon 8
4.2 kallisto versus RSEM and Iso-EM2 8
4.3 Miscellaneous kallisto statistics 9

5 Real wastewater data sequencing depth 10

6 Bootstrap analysis using kallisto 10

7 Genome coverage versus Ct values across the US 10

8 Further benchmarking experiments 14
8.1 The VLQ pipeline predictions are only mildly affected by uneven coverage (amplicon dropout) 14
8.2 No bias towards sequences overrepresented in the reference database 15
8.2.1 Experiment 1 15
8.2.2 Experiment 2 15

1 SARS-CoV-2 genomes show sequence diversity within variant lineages

We analyze within-lineage diversity for the reference set used for lineage quantification throughout the manuscript. This reference set was built by selecting representative genomes per lineage from the GISAID database[1], downloaded on 9 March 2021, of US origin (also see Methods in main paper). After removing low-quality sequences (defined as having less than 29,500 non-ambiguous nucleotides) we randomly selected 1000 sequences per lineage for further analysis. We used minimap2 and paftools to align each of these sequences to the reference genome (MN908947.3) and subsequently identify variation with respect to this reference. We then used VCFtools[2] to compute allele frequencies within each lineage. Figure S1 shows these allele frequencies for each variant lineage considered.

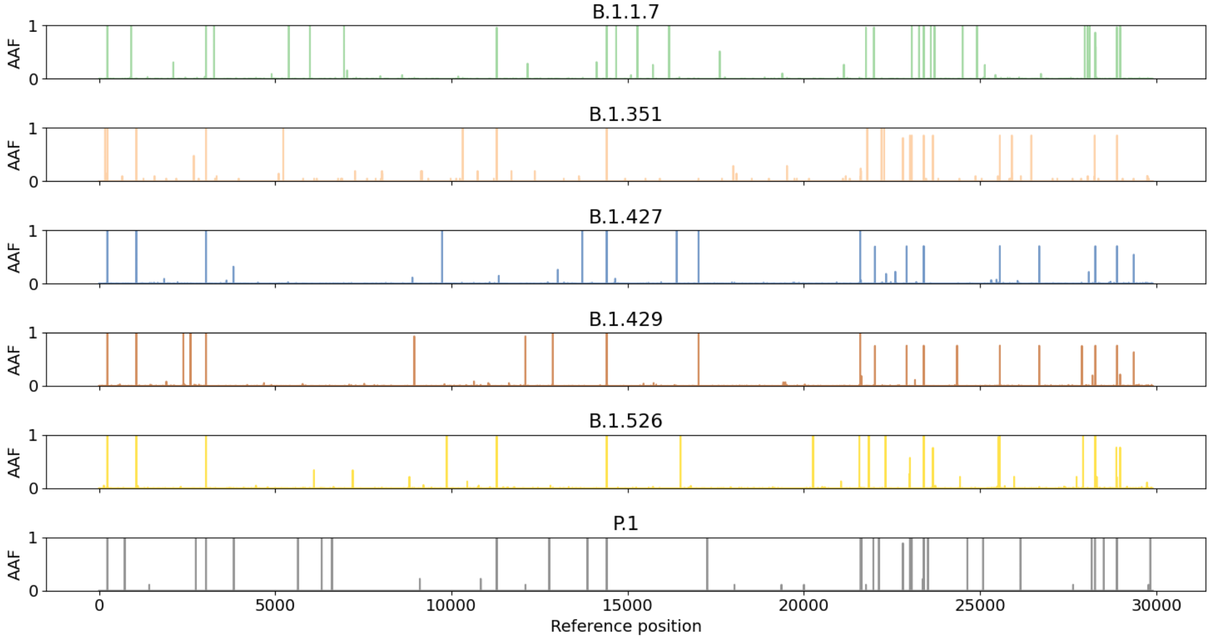

Figure S1: Within-lineage diversity observed in SARS-CoV-2 genomes on GISAID (downloaded 9 March 2021). The horizontal axis shows the position (in base pairs) on the reference genome (accession MN908947.3). The y-axis shows the alternative allele frequency (AAF), i.e. the fraction of genomes with a different nucleotide at a given position than the reference genome. This plot was computed by randomly selecting 1000 genomes of US origin per lineage.

[1] https://www.gisaid.org
[2] https://vcftools.github.io/index.html

2 Prediction noise level per sequence determines the minimal abundance threshold

We analyzed the noise level to determine a suitable minimal abundance threshold. This was determined experimentally: on a dataset which consists for 100% of B.1.1.7 sequences, we observe a noise level up to 1%, with most false positives below 0.1% (Figure S2). We therefore decided to apply a minimal abundance threshold of 0.1% per individual sequence in our reference set. Increasing this threshold would further denoise the predictions, but we may also lose valuable information. In particular, we would lose the ability to predict lineages of very low abundance. This is why we decided on a conservative threshold of 0.1%, but in practice anything between 0.1 and 1% would work. As we show in Table 1 of the main manuscript, increasing the threshold to 1% leads to higher precision at the cost of slightly lower recall compared to a threshold of 0.1%. In the end, the optimal choice depends on the application: if the purpose is to find lineages of low abundance, it is safer to use a conservative threshold of 0.1%, while a focus on predicting lineages at higher abundance would warrant a threshold of 1%. This is up to the user, the filtering threshold value can be specified when running our pipeline.

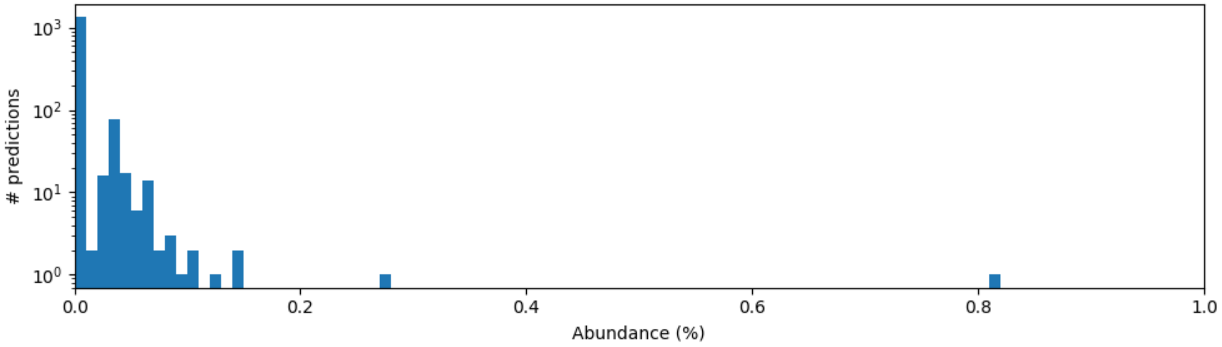

Figure S2: Histogram of raw abundances predicted by kallisto on a simulated dataset consisting of 100% B.1.1.7. The majority of false positives (background noise) can be filtered out by applying a minimal abundance threshold of 0.1%. True predictions occur at higher abundances (beyond the x-axis limit of 1.0%).

3 Benchmarking results for the VLQ pipeline

3.1 Full results for datasets main paper

Figure S3 shows the estimated variant abundances and relative prediction errors for the Spike-only 100x, Spike-only 1000x and whole genome 100x datasets that were described in the main manuscript.

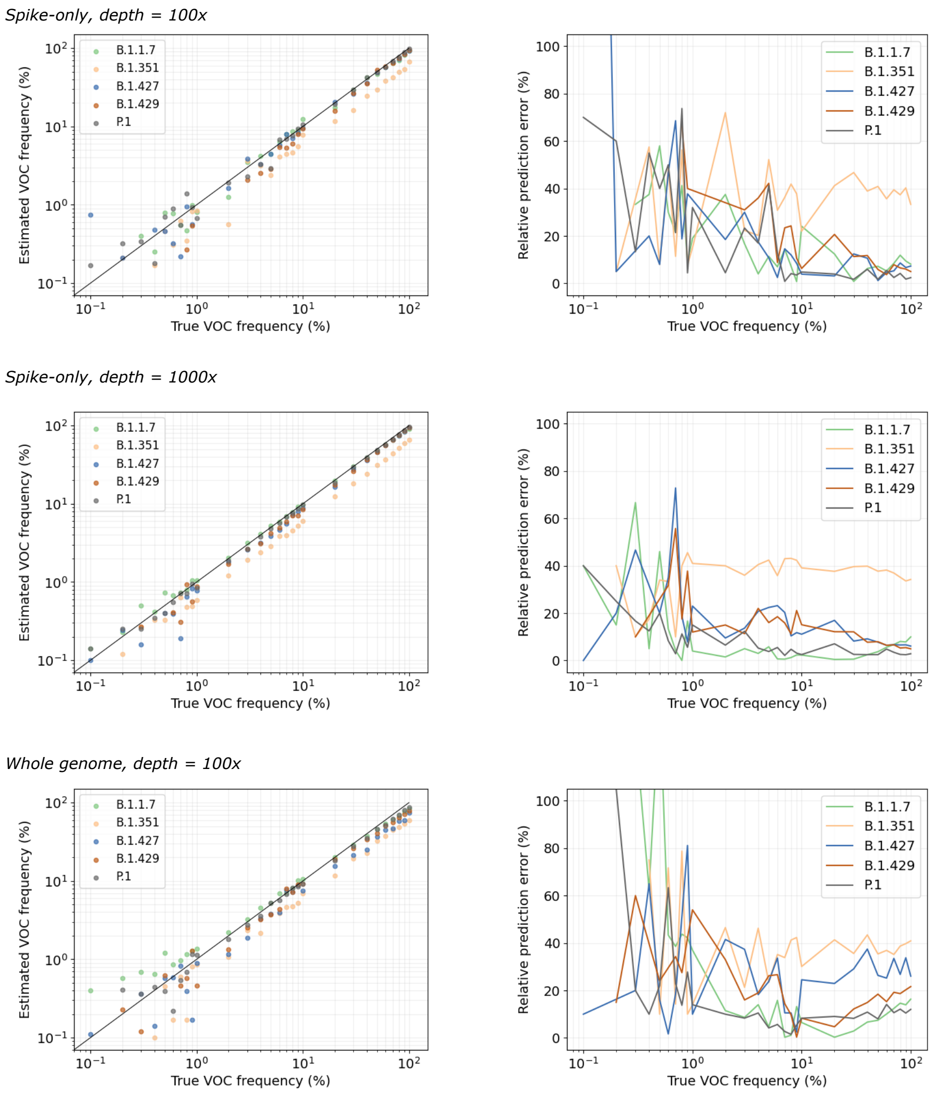

Figure S3: Estimated variant abundances and relative prediction errors for the Spike-only 100x, Spike-only 1000x and whole genome 100x datasets, respectively. Relative prediction errors are defined as the absolute difference between true and estimated frequency, relative to the true frequency.

3.2 Impact of sequencing errors on prediction accuracy

To investigate the impact of sequencing errors on prediction accuracy, we have generated a collection of benchmarking data sets with varying error rates. In this analysis we distinguish between substitution errors, insertion errors and deletion errors. For each of these categories, we generated a collection of datasets for 4 variant lineages (B.1.1.7, B.1.351, B.1.617.2 and P.1) with error rates ranging from 0.001% to 10%. The ART HiSeq 2500 model was used to simulate sequencing errors. The true variant frequency was kept constant at 10.8%, as well as the background lineages that together comprise the remaining 89.8% of the sample. We then ran our prediction pipeline on each of these datasets to estimate the relative abundance of the variant lineage. Figure S4 shows the resulting relative prediction errors per lineage for each error type (substitution, insertion and deletion errors, respectively).

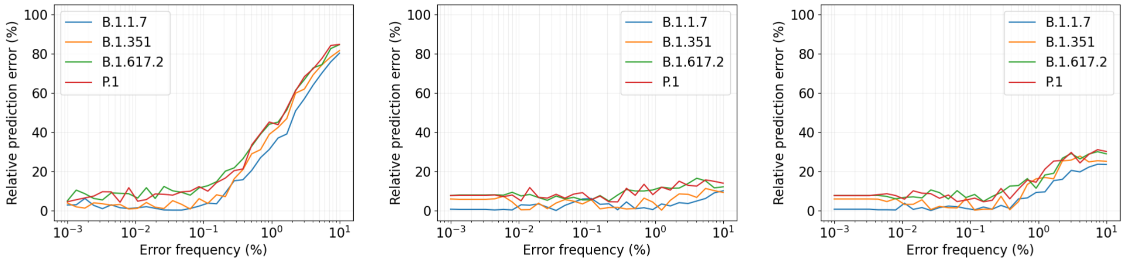

(a) Substitution errors (b) Insertion errors (c) Deletion errors

Figure S4: Relative prediction error (%) plotted against induced error frequency (%) for (a) substitution errors, (b) insertion errors, and (c) deletion errors. These experiments were done at a true VOC frequency of 10.8%.

We observe that insertion and deletion errors have very little impact on prediction accuracy, given that Illumina sequencing error rates are generally in a range of 0.1–1%. Substitution errors, however, do impact predictions as the error rate approaches 1%. The explanation for this is simple: due to high diversity between lineages, a substitution error will often match another lineage, thus leading to misclassification of the corresponding read and hence underestimation of the corresponding lineage. Figure S5 shows all data point for the substitution error experiment. We notice that substitution errors can explain the consistent underestimation we observe for our pipeline: with error-free reads, there is no underestimation (but we still see overestimation).

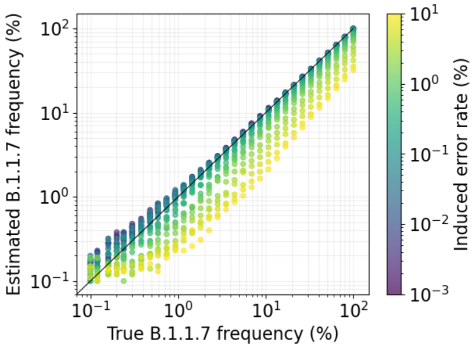

(a) B.1.1.7

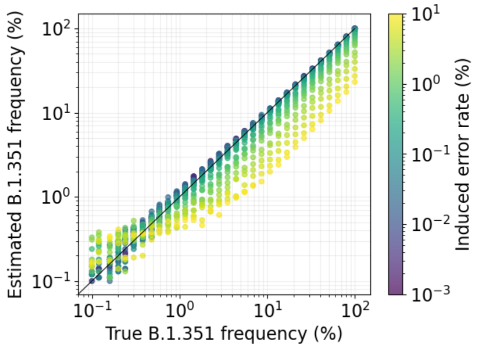

(b) B.1.351

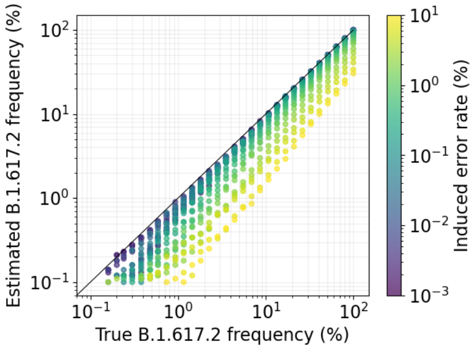

(c) B.1.617.2

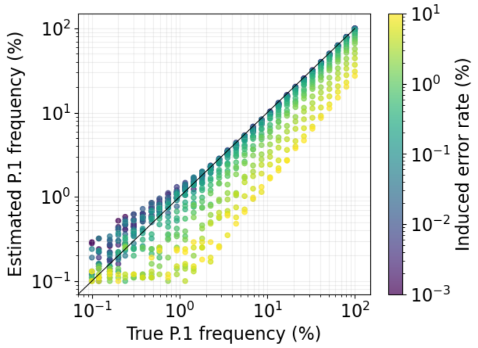

(d) P.1

Figure S5: All datapoints obtained for the substitution error datasets for (a) B.1.1.7, (b) B.1.351, (c) B.1.617.2 and (d) P.1. Predicted VOC frequency (%) is plotted against true VOC frequency (%). The substitution error rate is depicted by color (see color scale on the right). These experiments were done at a true VOC frequency of 10.8%.

3.3 The VLQ pipeline can distinguish between highly similar sequences

Since many lineages differ only by a couple of mutations, we investigate here to what extent we can distinguish between highly similar sequences. For the purpose of this experiment, we created artificial sequences by introducing random mutations (substitutions) in the original SARS-CoV-2 reference genome (MN908947.3). Through this procedure, we created 10 artificial lineages which differ in 3, 6, 9, ..., 30 positions from the reference genome, respectively. This corresponds to sequences with an edit distance compared to the reference genome of 0.01%, 0.02%, ..., 1%, respectively. Then, we simulated ten wastewater sequencing data sets, each of which contains the MN908947.3 reference sequence and one of the artificial mutants described above. Both sequences were simulated at 50% abundance. We ran our abundance estimation pipeline on each of these ten datasets to evaluate the ability of kallisto to distinguish between highly similar lineages.

Figure S6 shows the relative prediction error[3] for the original sequence as well as the simulated mutant. We observe that for each of these sequences the prediction error is close to zero, even at an edit distance of only 0.01%. This suggests that our pipeline can perform well even if the sequences contained in the wastewater data are highly similar.

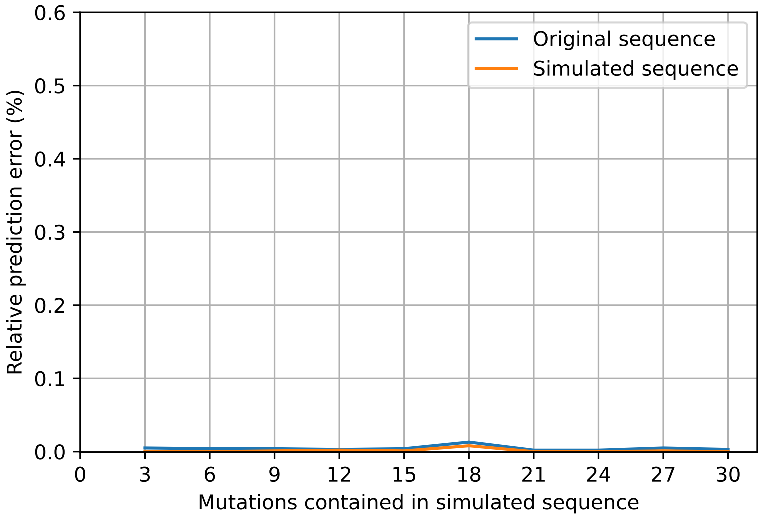

Figure S6: Relative prediction error (%) for a mixture of two lineages (original reference + artificial mutant) with increasing pairwise edit distance.

Note that the setting in this experiment is intentionally very simple: the reference set consists of all mutant sequences and the original reference sequence and the simulated wastewater data consists of only two sequences, equally abundant. This allows us to evaluate *only* the ability to distinguish between highly similar sequences without other complicating factors. While we conclude that kallisto can distinguish between highly similar sequences, in practice this will depend on factors like within-lineage diversity and the composition of the reference set in use. How to design an optimal reference set is an important question that is beyond the scope of this research.

[3] relative prediction error (%) = |predicted abundance − true abundance|/true abundance $*$ 100

4 Comparing kallisto to other RNA transcript quantification tools

To motivate our choice to use kallisto for predicting lineage abundance, we compare predictions to three other state-of-the-art tools for RNA transcript quantification: salmon, RSEM and Iso-EM2.

4.1 kallisto versus salmon

Since salmon uses a very similar RNA transcript quantification approach as kallisto, we started by comparing abundance estimates obtained with salmon and kallisto through our pipeline. We found that predictions by salmon were highly similar to those obtained with kallisto (Figure S7), the main difference being that salmon is slightly more conservative: it achieves higher precision (fewer false positives), at the expense of lower recall (more false negatives).

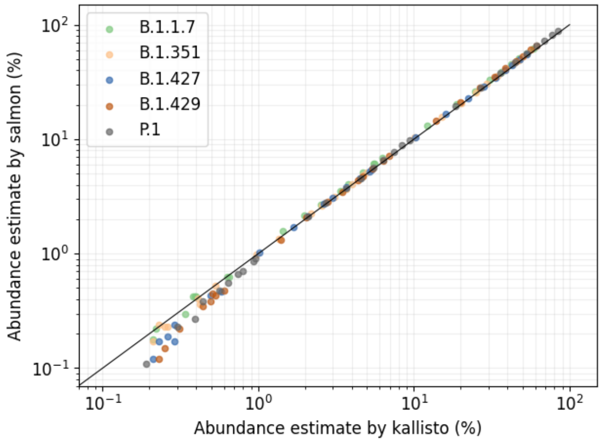

Figure S7: salmon versus kallisto abundance estimates per variant.

4.2 kallisto versus RSEM and Iso-EM2

We proceeded by comparing kallisto to two other RNA transcript quantification algorithms, RSEM[4] and Iso-EM2[5], which are also based on EM-algorithms. In terms of read alignment these methods follow a very different approach than kallisto and salmon: while salmon and kallisto use pseudoalignment to a de Bruijn graph, RSEM and Iso-EM2 use specialized read-to-transcriptome aligners. RSEM uses Bowtie (built-in) for read alignment and with Iso-EM2 it is up to the user to provide alignment files; for the experiments below, we used HISAT2[6].

We evaluate the transcriptome quantification tools (kallisto, salmon, RSEM, Iso-EM2) on a selection of 4 datasets where the variant lineage (B.1.1.7) is present at 1, 10, 50, 100%, respectively. Table S1 shows the prediction results, as well as runtime and peak memory usage.

An important parameter in HISAT2 (used to generate the alignments for Iso-EM2) is the -k parameter, which specifies the maximum number of alignments that HISAT2 will search for a given read. The default value is 10, but we believe that for our application a larger value may be necessary to achieve accurate predictions, since some regions of the SARS-CoV-2 genome have only little diversity. Hence, we ran HISAT2 with the default setting (-k 10) as well as a higher threshold (-k 100). Ideally, we would need -k 1000, but this was not feasible: memory usage exploded, as well as the size of the corresponding output file. Most likely, it

[4] `https://deweylab.github.io/RSEM/`
[5] `https://dna.engr.uconn.edu/?page_id=105`
[6] `http://daehwankimlab.github.io/hisat2/`

would not have been feasible for Iso-EM2 to process such a large output file either, as runtime and memory usage already increased substantially using -k 100 (Table S1).

Note that one of the major strengths of kallisto is that instead of keeping unnecessarily large SAM/BAM files, it only keeps compatibility classes. This is beneficial to runtime, memory usage and storage space needed. As a result, kallisto does not need to limit the number of hits allowed per read (contrary to read-to-transcriptome aligners), which benefits its prediction accuracy.

	abundance estimate (%)	absolute error (%)	relative error (%)	runtime (s)	memory (GB)
**true abundance = 1%**					
kallisto	1.4	0.4	40.0	148	1.7
salmon	2.6	1.6	60.0	13	0.7
Iso-EM2 - k10	1.1	0.1	10.0	141	19.2
Iso-EM2 - k100	1.3	0.3	30.0	960	35.2
RSEM	3.1	2.1	210.0	101	0.1
**true abundance = 10%**					
kallisto	10.6	0.6	6.0	177	1.7
salmon	15.3	5.3	53.0	13	0.7
Iso-EM2 - k10	4.1	5.9	59.0	132	18.8
Iso-EM2 - k100	6.1	3.9	39.0	1035	37.1
RSEM	18.0	8.0	80.0	154	0.1
**true abundance = 50%**					
kallisto	46.0	4.0	8.0	150	1.6
salmon	50.9	0.9	1.8	15	0.7
Iso-EM2 - k10	13.4	36.6	73.2	184	15.5
Iso-EM2 - k100	18.7	31.3	62.6	906	34.1
RSEM	56.0	6.0	12.0	153	0.1
**true abundance = 100%**					
kallisto	84.1	15.9	15.9	145	1.5
salmon	76.2	23.8	23.8	18	0.8
Iso-EM2 - k10	23.9	76.1	76.1	59	4.6
Iso-EM2 - k100	31.4	68.6	68.6	938	40.1
RSEM	80.8	19.2	19.2	183	0.2

Table S1: Abundance estimation errors, runtime and memory usage for kallisto, RSEM and Iso-EM2.

4.3 Miscellaneous kallisto statistics

To provide further insight into kallisto's performance, we provide some additional statistics for the simulated datasets analyzed above, as well as the three real datasets analyzed in Section 5. Table S2 presents the number and percentage of aligned reads, as well as the number and percentage of uniquely aligned reads. The latter is very low, since many sequences are highly similar.

	# aligned reads	% aligned reads	# uniquely aligned	% uniquely aligned
true freq = 1%	96023	97.0	2488	2.5
true freq = 10%	95896	96.9	2535	2.6
true freq = 50%	95896	96.9	2651	2.7
true freq = 100%	95859	96.8	2763	2.8
New Haven ER2	1873796	99.1	32636	1.7
New Haven EX1	1544959	98.8	12190	0.8
New Haven FH1	567384	99.0	4359	0.8

Table S2: Kallisto pseudoalignment statistics.

5 Real wastewater data sequencing depth

Wastewater sequencing data is extremely noisy and subject to severe amplification bias. We analyzed sequencing depth for the sequencing data obtained from New Haven (Connecticut, USA). Figure S8 shows the depth per genome position for a selection of three samples that represent samples with low, medium and high genome coverage, respectively. We notice that even for the sample with high genome coverage (99%), the sequencing depth differs between amplicons by orders of magnitude: some amplicons reach a sequencing depth of only 100x, while others reach a depth of more than 50,000x. Also for the other samples the differences in depth between amplicons are extreme, with many amplicons having 0 depth, while others reach 100,000x or more.

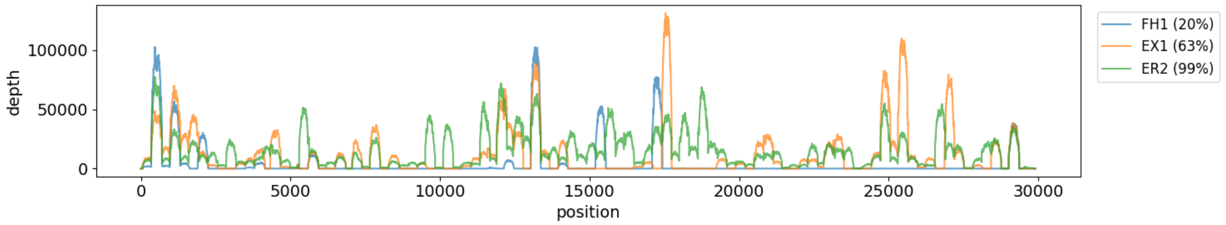

Figure S8: Sequencing depth along the genome for three samples collected in New Haven, CT. The first sample (FH1) has low genome coverage (20%), with very few amplicons reaching high sequencing depth. The second sample (EX1) has moderate genome coverage (63%), with roughly half of the amplicons reaching high sequencing depth. The third sample (ER2) has high genome coverage (99%), with nearly all amplicons reaching high sequencing depth.

6 Bootstrap analysis using kallisto

Kallisto offers a bootstrapping feature, through which the sequencing data is resampled at least 100 times and lineage abundances are predicted for each of these resampled datasets. The resulting predictions can subsequently be analyzed to obtain confidence intervals for the predicted abundance on the original dataset. We ran kallisto in bootstrap mode for all real sequencing data presented in the main manuscript, that is, the New Haven sludge samples (Figure S9) and the selection of samples from across the US (Figure S11).

7 Genome coverage versus Ct values across the US

Similar to the analysis for the New Haven sequencing data presented in the manuscript (Fig 3b), we analyzed genome coverage versus Ct values for a variety of samples from across the US (Figure S10). These samples were sequenced had slightly higher average Ct values than the sludge samples analyzed above (33.1 vs 32.3), indicating slightly lower RNA yield, and reduced genome coverage compared to sludge samples.

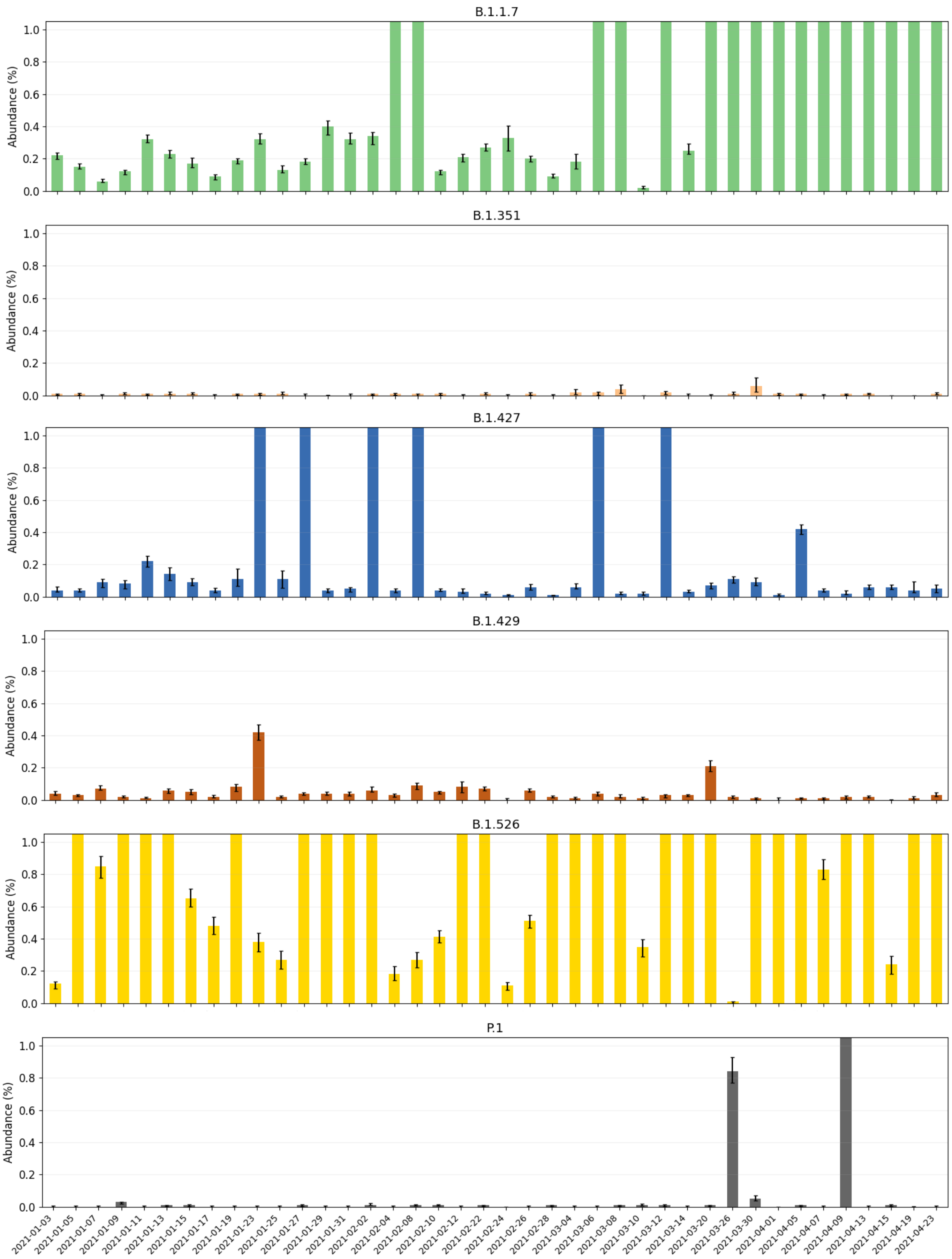

Figure S9: Raw predictions per variant with confidence intervals based on bootstrap analysis for New Haven samples. Note that in all subplots the y-axis is capped at 1% for improved readability.

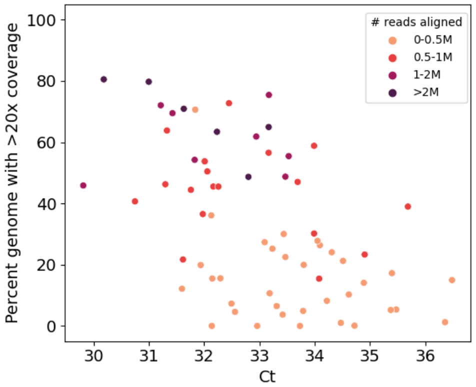

Figure S10: Percent genome coverage versus Ct values for samples across the US

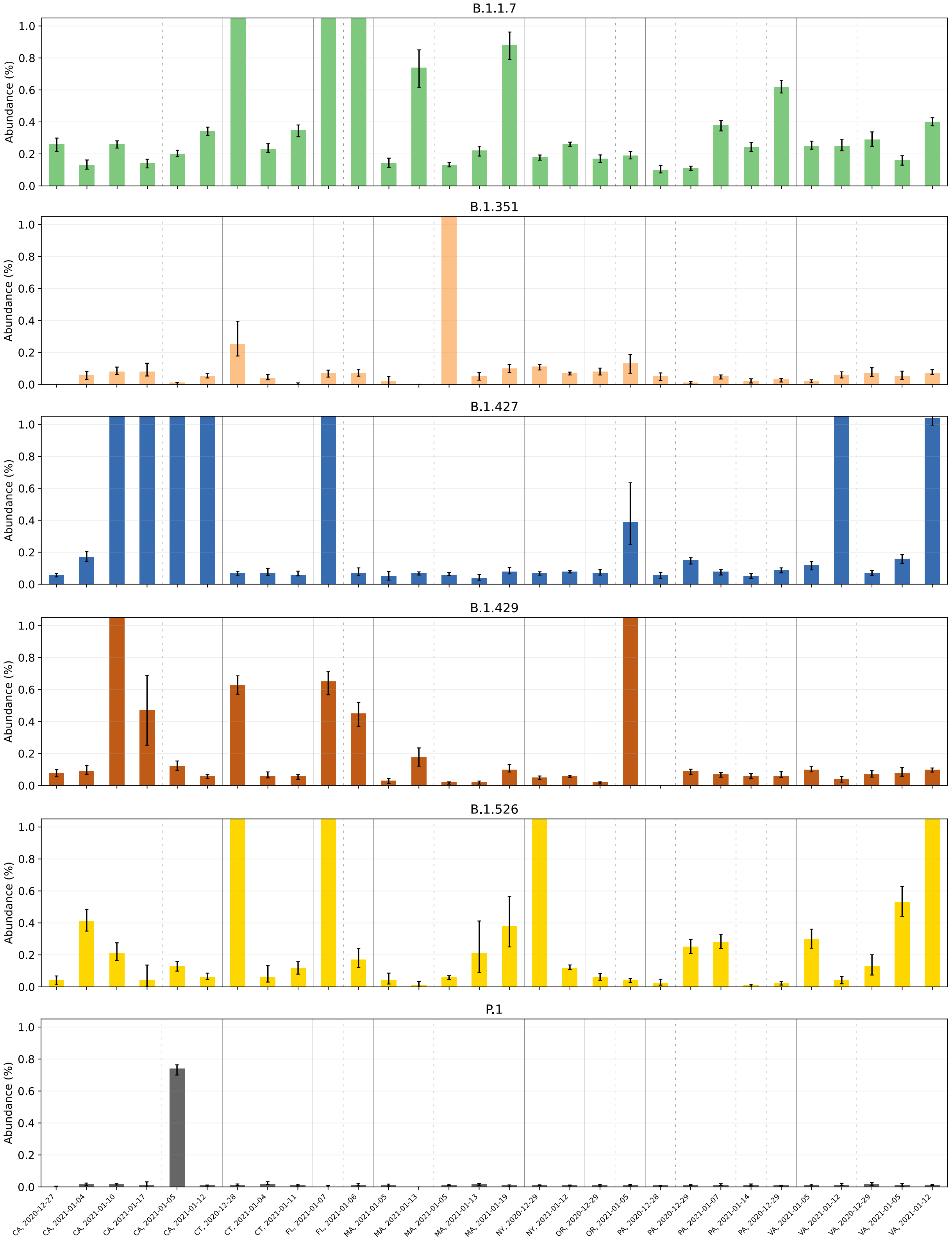

Figure S11: Raw predictions per variant with confidence intervals based on bootstrap analysis for samples across the US. Note that in all subplots the y-axis is capped at 1% for improved readability.

8 Further benchmarking experiments

8.1 The VLQ pipeline predictions are only mildly affected by uneven coverage (amplicon dropout)

While the benchmarking data presented in the manuscript already accounts for some variability in coverage, it does not reflect the extreme variability (amplicon dropout) observed in real wastewater sequencing data (Figure S8). Hence, we have created additional benchmarking datasets to evaluate the impact on our lineage abundance predictions.

These benchmarking data sets were created from the main benchmarking data sets presented in the manuscript (whole genome, 1000x, B.1.1.7 variant lineage at frequencies ranging from 0.1% to 100%). The reads were aligned to the SARS-CoV-2 reference genome and we divided the genome into regions of 400 bp (representing amplicons). Then, we downsampled reads per window to a fraction decided by a random probability generator that follows a beta distribution (parameters $\alpha = 0.5$ and $\beta = 0.5$). This results in a different downsampling rate per window to reflect varying sequencing depths across amplicons. The beta distribution used to decide the rate of downsambling favors extreme values (near 0 and 1), which results in amplicon dropout as observed in real wastewater sequencing data.

To account for the randomness in this downsampling procedure, we repeated this 10 times for each original dataset, thus producing 10 datasets per true VOC frequency. We ran our prediction pipeline on each of these datasets to estimate B.1.1.7 abundance. Figure S12 shows the mean abundance estimate per true VOC frequency, error bars represent the standard deviation among the 10 individual predictions per data point. We observe small error bars and the similar averages as for the original data (main manuscript), indicating that uneven coverage and random amplicon dropout has only a mild impact on the predicted abundances.

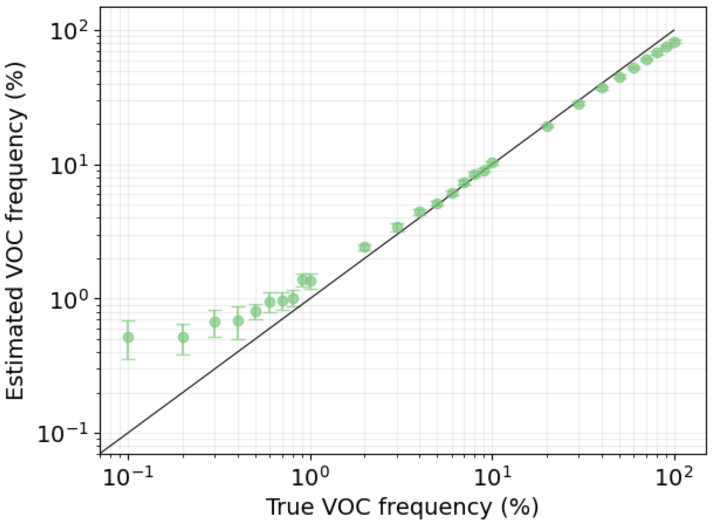

Figure S12: Estimated variant abundances for the whole genome 1000x datasets with B.1.1.7 as variant lineage, after downsampling per window of 400 bp to reflect uneven coverage and amplicon dropout in real wastewater sequencing data. Data points correspond to the mean abundance estimate per true VOC frequency, error bars represent the standard deviation among the 10 individual predictions.

8.2 No bias towards sequences overrepresented in the reference database

Since some of the lineages in our reference set have much more representative sequences in our reference set compared to others, we examined if overrepresented sequences are affected by estimation bias. First, we tested whether overrepresented lineages that are *not* in the simulated sequencing data are predicted to be present due to the fact that they are more likely to get reads assigned by chance (experiment 1). Then, we tested whether increasing the number of representative sequences for a given lineage in our reference set that is also present in the simulated wastewater data affects prediction accuracy (experiment 2).

8.2.1 Experiment 1

For this experiment, we created benchmarking datasets consisting of lineages C.37 and P.1, both at 50% abundance. Sequences were selected from GISAID for Connecticut in April 2021. Then, nine reference sets were created, each of which contains one sequence of the C.37 and P.1 lineage and 1, 2, . . . , 9 sequences of the B.1.1.7 lineage, respectively. The reference sequences were selected from GISAID for samples taken in March 2021 in the USA.

Figure S13 shows the relative prediction error for each of the two sequences present in the wastewater (C.37 and P.1), using reference sets with an increasing number of B.1.1.7 sequences. We observe that the increasing number of B.1.1.7 sequences in the reference set does not lead to abundance being falsely assigned to any of the B.1.1.7 sequences. Hence, we conclude from this experiment that there is no bias towards overrepresented sequences when the corresponding lineages are *not* present in the sequencing data.

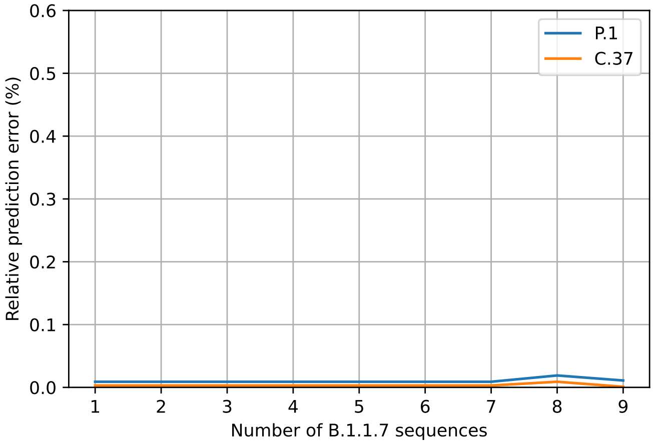

Figure S13: Relative prediction error (%) for the P.1 and C.37 sequences using reference sets with an increasing number of B.1.1.7 sequences.

8.2.2 Experiment 2

The design of this experiment is similar to the previous one, except here the sequences used to simulate wastewater sequencing data are BA.1 and BA.2 (Omicron sublineages). These sequences were sourced from Connecticut with collection date the 4th of February 2022. For the reference set construction, a full data set composed of BA.1 sequences from the USA was downloaded from GISAID[7] for the time period between the 21st of December and the 31st of December. During the reference set construction process 18 BA.1 sequences were selected. These 18 sequences were downsampled to 1, 2, . . . , 10 sequences, respectively, in order to

[7]`https://gisaid.org`

create 10 reference sets with an increasing number of BA.1 sequences. Each of these reference sets contains only a single BA.2 sequence, which was sampled in the USA in the same period.

Figure S14 shows the abundance estimates for BA.1 and BA.2 using the different reference sets with an increasing number of BA.1 sequences. The abundance estimates drastically improve after adding the second BA.1 sequence to the reference set (i.e. two BA.1 sequences in total) and remain almost perfectly equal to the true abundance.

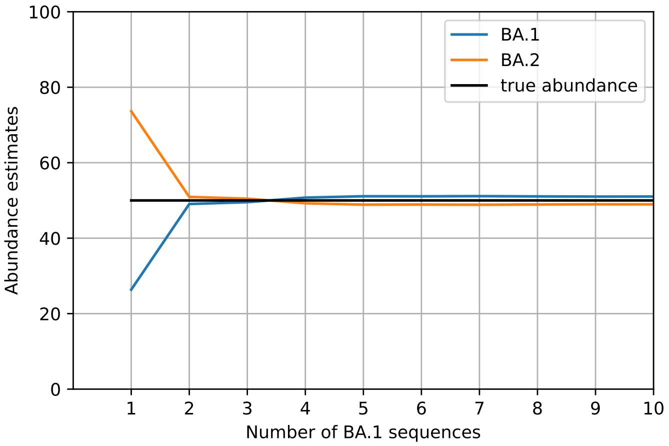

Figure S14: Abundance estimates per lineage per reference set, for reference sets with an increasing number of BA.1 reference sequences. True abundance is 50% for each lineage.

The table below shows the edit distance between each BA.1 reference sequence and the benchmark sequences BA.1 and BA.2. The second BA.1 sequence has the smallest edit distance and is therefore the most similar reference sequence to the BA.1 benchmark sequence. This explains why adding this sequence improves prediction accuracy substantially.

	Ref seq no 1	Ref seq no 2	Ref seq no 3	Ref seq no 4	Ref seq no 5	Ref seq no 6	Ref seq no 7	Ref seq no 8	Ref seq no 9	Ref seq no 10	Ref seq BA.2
BA.1	307.0	39.0	333.0	51.0	154.0	457.0	57.0	862.0	288.0	94.0	198
BA.2	394.0	164.0	455.0	162.0	180.0	448.0	122.0	922.0	290.0	154.0	132

Abundance estimates for BA.1 and BA.2 are both highly accurate, even though the reference set contains much more sequences for BA.1 than for BA.2. We also observe that prediction accuracy does not necessarily improve by adding more sequences to the reference set: what matters most is that the reference set contains sequences that represent the sample well. By adding more reference sequences, one increases the chances of providing a suitable reference sequence. We conclude that adding more sequences per lineage does not introduce estimation bias, but it does increase prediction accuracy.
